# Left ventricular epicardial lead placement after Carillon placement in the coronary sinus

**DOI:** 10.1007/s12471-019-1298-2

**Published:** 2019-07-01

**Authors:** C. A. da Fonseca, F. S. van den Brink, M. Feenema, K. Kraaier, T. N. Vossenberg

**Affiliations:** Medical Center Leeuwarden, Leeuwarden, The Netherlands

**Fig. 1:**
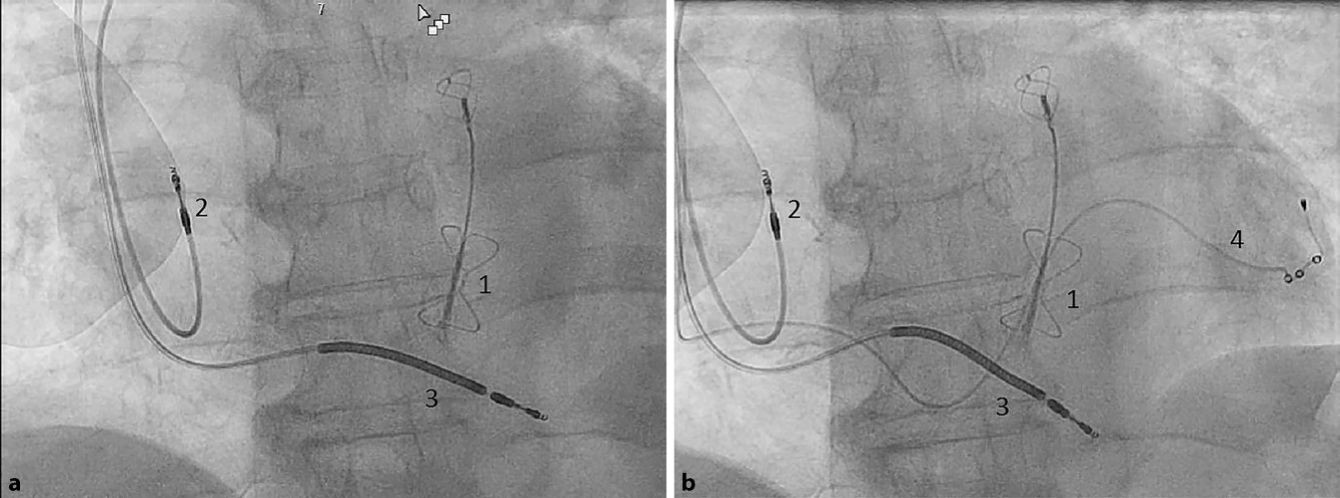
**a** The Carillon device (*1*) with the right atrial (*2*) and right ventricular (*3*) leads; **b** the Carillon device (*1*) and the left ventricular epicardial lead (*4*) in the coronary sinus. This illustrates the possibility of placing a Carillon device and a left ventricular lead in the coronary sinus in the treatment of mitral regurgitation and heart failure

A 72-year-old man with ischaemic cardiomyopathy and secondary mitral regurgitation developed heart failure. Due to the absence of options for revascularisation that would improve left ventricular function, poor functional status and a high Euro-SCORE II, the patient was deemed unfit for surgery. Furthermore, a cleft mitral valve also made him unsuitable for MitraClip [1]*.* To reduce mitral regurgitation he received treatment with a Carillon device for mitral valve annuloplasty in the coronary sinus (Fig. 1; [2]). Unfortunately, this did not reduce MR. In time he developed a left bundle branch block which made him eligible for placement of a cardiac resynchronisation therapy defibrillator (CRT-D) [3].

There were no complications and the patient’s functional status improved significantly.
